# Supplementary material for: Splice-Junction-Based Mapping of Alternative Isoforms in the Human Proteome
Source: Cell Rep. Author manuscript; Available in PMC 2020 Jan 15. (PMC6961840; doi:10.1016/j.celrep.2019.11.026)
Supplement: 2 [file NIHMS1546469-supplement-2.zip › DF1/PXD006675/PulmonaryArtery/PulmonaryArtery_10_CALD1_HTENTFSVPDEEAKTTTTNTQVEGDDEAAFLER.pdf]

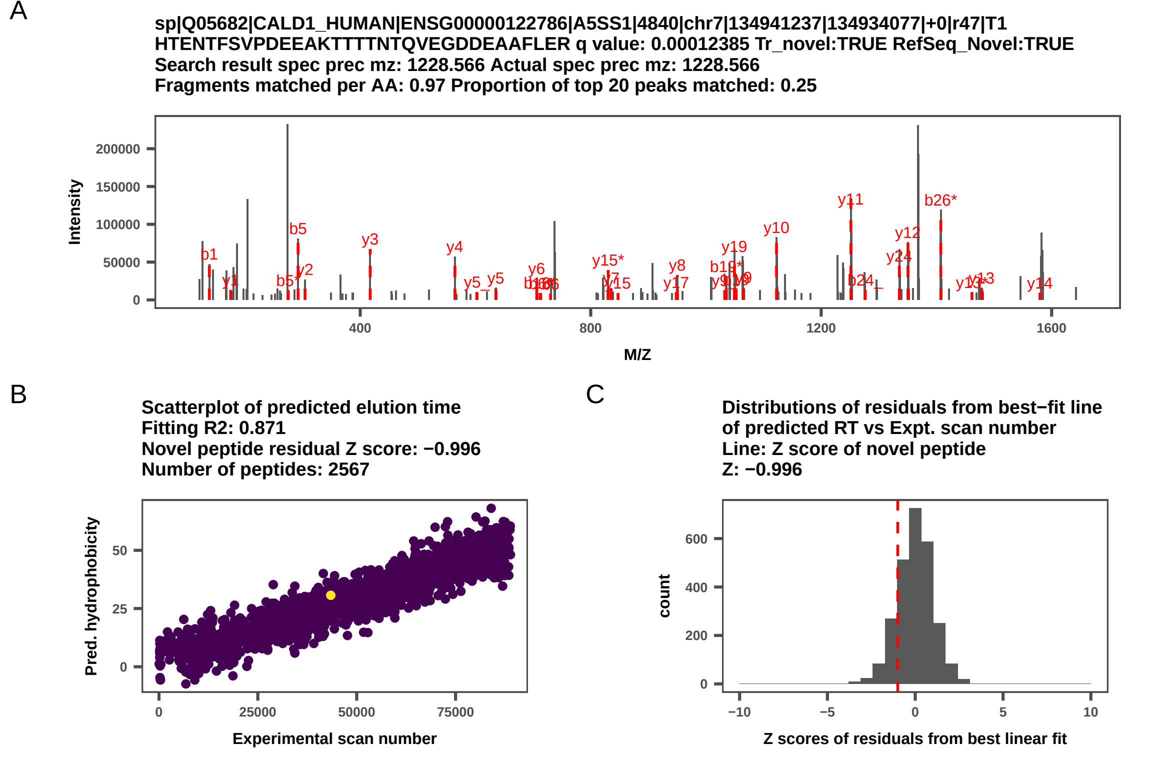
A
sp|Q05682|CALD1_HUMAN|ENSG00000122786|A5SS1|4840|chr7|134941237|134934077|+0|r47|T1
HTENTFSVPDEEAKTTTTNTQVEGDDEAAFLER q value: 0.00012385 Tr_novel:TRUE RefSeq_Novel:TRUE
Search result spec prec mz: 1228.566 Actual spec prec mz: 1228.566
Fragments matched per AA: 0.97 Proportion of top 20 peaks matched: 0.25
Intensity
200000
150000
100000
50000
0
b1
y1
b5
b5*
y2
y3
y4
y5
y6
y15*
y15
y8
y17
b19*
y19
y10
y11
b24
y12
y24
b26*
y14
400
800
1200
1600
M/Z
B
Scatterplot of predicted elution time
Fitting R2: 0.871
Novel peptide residual Z score: −0.996
Number of peptides: 2567
Pred. hydrophobicity
50
25
0
0
25000
50000
75000
Experimental scan number
C
Distributions of residuals from best−fit line
of predicted RT vs Expt. scan number
Line: Z score of novel peptide
Z: −0.996
count
600
400
200
0
−10
−5
0
5
10
Z scores of residuals from best linear fit
